# Men’s Gender Norms and Gender-Hierarchy-Legitimizing Ideologies: The Effect of Priming Traditional Masculinity Versus a Feminization of Men’s Norms

**DOI:** 10.1007/s12147-022-09308-8

**Published:** 2023-01-16

**Authors:** Giulia Valsecchi, Vincenzo Iacoviello, Jacques Berent, Islam Borinca, Juan M. Falomir-Pichastor

**Affiliations:** 1 Department of social Psychology, FPSE, Université de Genève, Uni Mail, Boulevard du Pont d’Arve 40, CH-1205 Geneva, Switzerland; 2 School of Psychology, University College Dublin, Dublin, Ireland

**Keywords:** Precarious manhood, Social role theory, Traditional gender norms, Men’s gender norms feminization, Gender-hierarchy-legitimizing ideologies

## Abstract

Contemporary evidence suggests that masculinity is changing, adopting perceived feminine traits in the process. Implications of this new masculine norm on gender relations remain unclear. Our research aims to better understand the influence of changing masculine norms on men’s endorsement of gender-hierarchy-legitimizing ideologies. Based on Precarious Manhood Theory and Social Role Theory, we conducted two quasi-experimental studies (*N* = 412) in which we first assessed heterosexual men's motivation to protect traditional masculinity. Then, we informed them that men’s gender norms are becoming more feminine (feminization norm condition) or are remaining masculine in a traditional sense (traditional norm condition). In the third (baseline-control) condition, participants received no information about men’s gender norms. Finally, we assessed the extent to which participants endorsed gender-hierarchy-legitimizing ideologies, namely sexism (Study 1) and masculinist beliefs (Study 2). Results showed that men who were less motivated to protect traditional masculinity were less likely to endorse gender-hierarchy-legitimizing ideologies when exposed to the feminization and control conditions compared to the traditional norm condition. The implications of these findings for gender equality and gender relations are discussed.

According to masculine ideology, masculinity is a cultural construct defined by a set of beliefs and expectations regarding how men should behave in a given time and culture [58, 75]. The predominant contemporary masculine ideology in Western societies is referred to as traditional masculinity [9]. This ideology sustains men’s power over women by putting forward the idea that boys and men should be dominant, heterosexual, physically strong, and should avoid feminine behaviors and attitudes [9]. Indeed, endorsement of traditional masculinity is related to higher endorsements of sexist ideologies [42, 70, 71], negative attitudes toward gender equality [69, 89], sexual aggressivity [53, 81], and the belief that masculinity is expressed by sexual performance [80]. In other words, traditional masculinity ideology serves to justify and maintain traditional gender roles that sustain the gender hierarchy.

Although traditional masculinity seems to remain the predominant masculinity ideology nowadays [64], or at least men perceive it as such [52], recent evidence suggests that men’s role in Western societies is undergoing a transformation, adopting perceived feminine traits in the process. This transformation includes changes in men's traits, professional occupations, family dynamics, and the institutionalization of the role of men in the struggle for gender equality [49, 67]. While the effects of changes in masculinity may be slow and unnoticed in some contexts [5, 21], people generally perceive that masculinity norms are changing [25, 66]. This perceived trend toward a feminization of the male gender norm raises the question of its consequences on gender relations. For example, this social change could ultimately increase gender equality, as it could free men from the prescriptions of traditional masculinity that constrain them into avoiding anything feminine-related (i.e., the antifemininity mandate). However, research has insufficiently investigated the consequences of this perceived feminization on intergroup relations. Indeed, to our knowledge, the only research investigating the influence of changing gender norms on men's attitudes toward non-traditional gender roles examined the perception that men and women are similar in agentic (or stereotypically masculine) traits [59]. No research has directly investigated how the perception that men’s norm is becoming more feminine impacts men's attitudes toward gender equality. To this end, the present research makes a novel contribution to the literature by experimentally testing the influence of the perceived feminization of the male gender norm compared to a traditional norm on men’s gender-hierarchy-legitimizing ideologies as a function of their motivation to protect traditional masculinity.

## Gender Norms and the Prevalence of Gender-Hierarchy-Legitimizing Ideologies

The norms defining traditional masculinity are hegemonic, meaning that they legitimize and uphold men’s privileged status in society and male dominance over women and subordinated masculinities, such as gay and racialized men [19, 20]. Central to the definition of the traditional male identity is the antifemininity mandate [13], which captures the construction of masculinity in opposition to femininity, and prescribes men to avoid attitudes, roles, and behaviors that reflect femininity [15, 48, 82].

Contexts characterized by prevailing traditional gender norms, and in particular traditional masculinity norms, are the least egalitarian with respect to gender relations (e.g., [12, 60, 61, 63, 76, 85]). For instance, cross-national studies show that countries where expectations about boys and men are more traditional register higher rates of violence against women [47], lower rates of work-life balance [38] and lower ratios of supportive laws to advance gender equality and women’s rights [86]. Moreover, in these countries, men are more prone to endorse stereotyped views of gender [72, 85], express more sexism [85] and less positive parenting behaviors [76]. Experimental research corroborates these findings and shows that priming a misogynistic or paternalistic norm among men decreases their likelihood to intervene against sexual harassment [63] and increases misogynistic cognitions—that is, cognitive networks related to insulting terms against women—among high-sexist men [12]. Taken together, cross-national surveys and experimental studies demonstrate that traditional masculinity norms are related to gender-hierarchy-legitimizing ideologies that subordinate women and legitimize men’s privileged status in society.

However, gender roles are being challenged, and traditional norms of masculinity (albeit slowly) are giving way to more progressive and feminized norms of masculinity [38, 86]. Indeed, evidence form Wester countries suggests that men’s role in society is evolving and that men are now taking on and expected to exhibit more caring and nurturing roles, once reserved uniquely for women. For example, men are getting more and more involved in unpaid work, especially in domestic activities [18, 29, 62, 73] such as the care of children [38]. Similarly, they are increasingly perceived as possessing traditionally feminine traits (e.g., [23, 35] and engaging in traditionally feminine activities [11]. Moreover, stereotypically feminine traits, such as empathy, communication and collaboration, are gaining value in typically masculine professional positions, such as leadership and management [e.g., 43]. These changes suggest that men are increasingly adopting roles that were traditionally reserved for- and/or perceived to be for women and seem to generate the overall perception that a new, more feminized norm of masculinity is emerging ([66, 67]; see also [35]). Consequently, this perceived feminization of men’s gender norms may challenge the antifemininity mandate central to the traditional definition of masculinity and may ultimately influence gender relations. To this end, in the present work, we are interested in investigating whether the perceived feminization of men’s gender norms (compared to traditional norms) influences men’s endorsement of gender-hierarchy-legitimizing ideologies.

## The Potential Consequences of the Feminization of Men’s Gender Norms (vs. a Traditional Norm)

Despite the theoretical and practical relevance of social changes in gender norms for gender equality, research examining the consequences of the feminization of men’s gender norms on men’s attitudes and behaviors toward women are scarce, and results are mixed. Indeed, to date, research examining the consequences of the feminization of men’s gender norms has focused solely on men’s self-description [3, 10, 13] and on backlash against non-normative ingroup members [36, 50, 51, 84]. The only research investigating the effects of a change in gender norms on intergroup relations emphasized men’s and women’s similarity in agentic traits, which are typically associated with traditional male norms ([59], Study 3). This condition of gender similarities was, therefore, more likely to elicit a representation of a masculinization of women’s gender norms rather than a feminization of men’s gender norms (see [21, 28]). Thus, research is still needed in order to examine specifically whether the feminization of men’s gender norms have direct consequences on gender relations.

In order to anticipate the potential consequences of a feminization of men’s gender norms (vs. traditional norms), we were informed by two theories in the domain of gender norms and behaviors: Social Role Theory (SRT; [30, 31]; see also [6]) and Precarious Manhood Theory (PMT; [88]). These two theoretical perspectives lead to opposing predictions, either with positive consequences for gender equality—that is, a general conformity toward more feminized norms of masculinity—or with negative consequences for equality—that is, a general resistance to this social change.

According to SRT ([30, 31]; see also [6]), the differences in traits and behaviors between men and women result from a socialization process based on the different distributions of men and women in social roles. The homemaker-provider model is the traditional role model more common in Western societies (at least until the feminist deconstruction of gender roles; [17, 26]. Each role is associated with communal (interdependence) or agentic (independence and assertiveness) traits ([1]; see also [22], which appear to enable individuals to perform these specific roles successfully [4]. Because women are more likely to occupy the homemaker role than men, they are believed to possess more communal traits. Conversely, because men are more likely to occupy the provider role than women, they are believed to possess more agentic traits [30, 34]. Important for the purpose of the present research is the idea of the malleability of social roles [24, 27, 41, 66]. Because gender roles are rooted in the division of labor, and the homemaker-provider model in particular, changes in the social structure should induce changes in gender roles and expectations.

Initial empirical evidence for this theoretical approach is provided by Eagly and Steffen [33]. In one study, participants were asked to judge women and men whose occupations were either homemaker (communal traits), full-time employee (agentic traits), or were not indicated. When the target occupation was not indicated, results supported the general hypothesis that the target’s stereotypes and traits derive from the gender division of labor: Women were perceived as more communal than agentic, while men as more agentic than communal. However, when occupation information was provided, it had a stronger impact on the target’s perception than the target's gender. Indeed, female and male homemakers were both equally perceived as being more communal than agentic, whilst female and male employees were both equally perceived as being more agentic than communal. This theoretical understanding is also consistent with findings based on System Justification Theory (SJT; [56], according to which people are motivated to defend and legitimize the systems in which they operate. For instance, participants rate women in business more positively and are more likely to agree that there should be more women in business when they are informed that women are well-represented (the many-women condition) rather than underrepresented (the few-women condition) in high-level business positions [57].

Overall, these findings are consistent with SRT’s predictions and suggest that men's and women's expectations change as their perceived representation in social roles changes [26, 32]. Accordingly, there are reasons to think that the perceived feminization of men’s gender norms will decrease the salience of the antifemininity mandate and then decrease men’s endorsement of gender-hierarchy-legitimizing ideologies. Conversely, priming a traditional norm should enhance the salience of the antifemininity mandate and increase men’s endorsement of gender-hierarchy-legitimizing ideologies.

Different predictions emerge based on PMT [87, 88]. PMT argues that manhood is an achieved social status that must be earned and can be easily lost. Unlike womanhood, which is mainly the result of physical and biological maturation and is hardly questioned once acquired [44], manhood is something that boys and men must (socially) earn and perform through their behaviors in order to be considered as ‘real men’. Consequently, and as compared to femininity, manhood can be lost more easily through social transgressions, such as enacting counter-stereotypically feminine behaviors or failing to demonstrate adequate levels of masculinity. Therefore, men respond to threats to their individual masculinity (i.e., prototypicality threat) and threats to the antifemininity mandate (i.e., ingroup distinctiveness threat) by engaging in compensatory behaviors in order to reaffirm their own masculinity ([14, 88] and/or to restore the antifemininity mandate [13], Study 5), respectively. In addition, it is worth noting that this type of response is consistent with the reactive-distinctiveness hypothesis, according to which ingroup members react to threats to group distinctiveness by strengthening intergroup differences [54, 55]. Thus, in line with PMT, priming a traditional norm should reassure men and decrease (or have no influence) on men’s endorsement of gender-hierarchy-legitimizing ideologies, whereas the feminization of men’s gender norms should challenge the antifemininity mandate and the ingroup distinctiveness, and trigger men’s endorsement of gender-hierarchy-legitimizing ideologies. This claim is supported by research showing that, when exposed to a condition emphasizing a decline in gender differences, as compared to a condition highlighting gender differences, male participants are more motivated to perform manhood-restoring behaviors [13],Study 5), express more sexual prejudice, and report more discomfort with homosexuality [36, 50, 84].

## Reconciling Both Theories: The Moderating Role of Motivation to Protect Traditional Masculinity

We believe that men’s personal motivation to protect traditional masculinity allows us to reconcile the opposing predictions derived from PMT and SRT and to explain in which circumstances the dynamics of conformity or resistance will be observed. Indeed, the motivation to protect traditional masculinity provides insight into men’s motivation to protect the gender status quo and men's privileged position in the gender hierarchy.

The feminization of men's gender norms translates into men adopting feminine roles and behaviors and implies that the norms defining masculinity now suggest that men can and should assume roles and behaviors once considered to be feminine. This directly challenges the antifeminine mandate of traditional masculinity, which is essential to justify and maintain the gender status quo. That said, a lower motivation to protect traditional masculinity implies a lower motivation to defend the antifemininity mandate when it is challenged. Consequently, when confronted with the feminization of their gender group, men who are less motivated to protect traditional masculinity should be more likely to conform to new, more feminized norms of male gender identity.

Conversely, stronger motivation to protect traditional masculinity implies a higher motivation to protect the antifemininity mandate when it is challenged. This higher motivation should lead to defensive reactions in response to the feminization of men’s gender norms aimed at restoring traditional masculinity and ensuring positive distinctiveness.

Empirical evidence informs us about the importance of motivation to protect traditional masculinity when considering men's reactions to changes in their gender norms. On the one hand, priming men who are less motivated to protect traditional masculinity with a feminization of their gender norms (vs. traditional norms) reduced their fear of being perceived as gay, their discomfort while imagining performing feminine behaviors [10], and the usage of traditional masculine traits in their self-descriptions (i.e., [3]. On the other hand, priming men who are strongly motivated to protect traditional masculinity with a feminization of their gender norms (vs. traditional norms) triggered defensive reactions aimed at restoring a self-perception as being a traditional man ([36, 84]; see also [3, 13], Study 5).

## The Present Research

The present research aims to investigate the consequences of the feminization of men’s gender norms versus a traditional norm on men’s gender-hierarchy-legitimizing ideologies as a function of their personal motivation to protect traditional masculinity. To test this, we conducted two quasi-experimental studies using two samples of heterosexual men. In both studies, we initially assessed participants’ endorsement of traditional norms of masculinity to assess their motivation to protect traditional masculinity. We then manipulated men’s gender norms (feminized, traditional, or control): In the *traditional* condition, we highlighted the normativity of the traditional traits and roles of masculinity among men, as well as the related strong gender differences. In the *feminized* condition, participants learned that men are becoming more feminine and that gender differences are blurring. The *control* condition did not include any information about the ingroup norm, which allowed us to determine where the baseline is located. Indeed, among past studies, only Bosson and Michniewicz [13] introduced a control condition and showed that the baseline lies between the conditions emphasizing a decline and an increase in gender differences. Consistent with this result, we expect the control condition to lie between the two experimental conditions manipulating men’s gender norms.

Finally, we assessed participants’ endorsement of gender-hierarchy-legitimizing ideologies as the main dependent variable. Based on PMT [87, 88] and SRT [31], we expected to observe a significant difference between the feminization and traditional conditions, with the control condition lying in between. However, whether the feminization (vs. traditional) norm increases or decreases gender-hierarchy-legitimizing ideologies should depend on men's motivation to protect traditional masculinity: The feminization of men’s gender norms (vs. traditional norms) should decrease gender-hierarchy-legitimizing ideologies among those who are less motivated to protect traditional masculinity (H1), while it should increase gender-hierarchy-legitimizing ideologies among those who are strongly motivated to protect traditional masculinity (H2). The baseline control condition should fall between the two experimental conditions.

### Study 1

In Study 1, we assessed ambivalent sexism [47] as a hierarchy-enhancing legitimizing ideology that justifies and upholds inequalities between men and women [77].

## Method

### Participants and Design

Based on recommendations to recruit at least 50 participants per experimental condition [78, 79], we recruited 233 heterosexual British men via Prolific (*M*_age_ = 44.99 years, *SD*_age_ = 15.18). Participants were invited to participate in an online survey and were compensated with £1 for their time. Because the entire sample agreed to the usage of their data, no participant was excluded from the analyses. A sensitivity power analysis conducted on G*Power for a multiple linear regression model with five predictors, assuming an α of 0.05, and power of 0.80, revealed that our final sample enabled us enough power to detect a small effect size (*f*^2^ = 0.05). The majority of participants identified as White British (89.7%; 3.9% identified as Asian British, 4.3% identified as Black British, and 2.1% identified as other).

Both studies were presented as “investigating men's opinion on several social issues”. We asked for demographic information at the beginning of the questionnaire. Participants completed the measures in the listed order and all response-scales ranged from 1 (*Not at all*/*Strongly disagree*) to 7 (*Absolutely*/*Strongly agree*). At the end of the survey, participants were thanked and debriefed. Both studies followed APA ethical guidelines and were approved by the ethics committee of the first author's institution. Materials and data for blind peer review of the two experimental studies are available at https://osf.io/jztdb/?view_only=33c96b83ea6146d68a5903d1ba96872b.

### Independent Variables

*Motivation to protect traditional masculinity* was assessed by measuring participants’ endorsement of traditional masculinity norms with the 26-item Male Role Norms Scale (MRNS; [82]. A global score was computed by averaging participants’ responses to the 26 items, whereby higher scores reflect a higher endorsement of traditional masculinity (*M* = 3.74, *SD* = 0.95, α = 0.92).

#### Manipulation of the Gender Norm

The experimental manipulation of men’s gender norms was induced as in recent research [51]. Participants in the traditional and feminization conditions were presented with a bogus article summarizing the results of a longitudinal study about the evolution of masculinity, the aim of which was to investigate men’s personality and behaviors from 1957 to 2017. The feminization condition [traditional condition in brackets] stated that “the results of the study suggest that men tend to become more feminine over time [are just as masculine as ever] and that the distinction between ‘being a man’ and ‘being a woman’ tends to disappear [remains fundamental]”. In order to reinforce the effect of the experimental manipulation, participants were asked to provide an everyday example that would corroborate the study’s findings. In the control condition, no information was provided.

### Measurements

#### Ambivalent Sexism Inventory

After being exposed to men’s norm manipulation, participants were asked to report their level of agreement with the 22-item Ambivalent Sexism Inventory [46]. The hostile sexism subscale (e.g., “Most women interpret innocent remarks or acts as being sexist”) and the benevolent sexism subscale (e.g., “Women should be cherished and protected by men”) were positively correlated, *r*(233) = 0.37, *p* < 0.001. After recoding, we computed a global score of ambivalent sexism by averaging participants’ responses to the 22 items. Higher scores reflect a higher endorsement of ambivalent sexism (*M* = 4.02, *SD* = 0.70, α = 0.80).

#### Experimental Manipulation Check

In order to check participants’ comprehension of the experimental manipulation, at the end of the study, participants were asked to indicate whether, in their personal opinion, “Men’s behaviors have changed in recent years,” “Men’s way of being has changed in recent years,” and “Today, men are more feminine than ever.” An overall score was computed by averaging the three manipulation check items, wherein higher scores reflect an acknowledgment of men’s feminization (*M* = 4.77, *SD* = 1.19, α = 0.83).

## Results

Regression analyses were conducted to test our hypothesis. The three levels of the manipulated variable were broken down into two contrasts.^1^ According to our hypotheses, the critical contrast tested the linear effect between the three experimental conditions by opposing the feminization norm condition to the traditional norm condition with the baseline-control condition situated in-between (C1: − 1 = traditional, 0 = control, + 1 = feminization), and the orthogonal contrast tested the residual variance by opposing the two norm conditions to the control condition (C2: − 1 = traditional and feminization, + 2 = control). According to the main hypothesis, we expect C1 to be significant and C2 to be not significant. We regressed the dependent variables on the two contrasts, motivation to protect traditional masculinity (standardized scores), as well as the interaction between motivation to protect traditional masculinity and each contrast (interactions between the two contrasts were not included).

### Experimental Manipulation Check

The analysis revealed a significant main effect of C1, *B* = 0.30, 95% *CI* = [0.11, 0.48], *t*(227) = 3.11, *p* < 0.01, η^2^_p_ = 0.04. Overall, acknowledgment that men’s gender norms are becoming more feminine was stronger in the feminization condition (*M* = 5.03) than in the traditional condition (*M* = 4.44), with the baseline-control condition (*M* = 4.83) lying in between the two experimental conditions. No other effect reached significance, *ts*(227) < 1.55, *ps* > 0.12. Simple effects revealed that the control condition did not differ from the feminization condition (*t*(173) = 1.07, *p* = 0.30) but differed from the traditional condition (*t*(173) = − 2.05, *p* = 0.04).

### Ambivalent Sexism Inventory

**Fig. 1:**
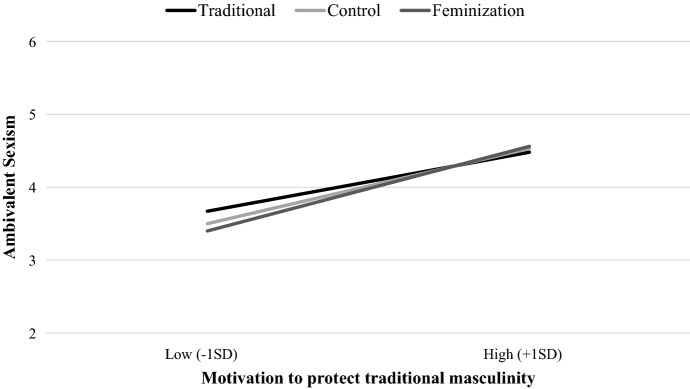
Effects of men’s gender norms on ambivalent sexism as a function of participants’ motivation to protect traditional masculinity (Study 1)

Analyses revealed a significant main effect of motivation to protect traditional masculinity, *B* = 0.51, 95% *CI* = [0.45, 0.57], *t*(227) = 15.55, *p* < 0.001, η^2^_p_ = 0.52. Overall, sexism increased as a function of men’s endorsement of traditional masculinity norms. This effect was qualified by a significant C1 × motivation to protect traditional masculinity interaction, *B* = 0.10, 95% *CI* = [0.02, 0.18], *t*(227) = 2.44, *p* = 0.016, η^2^_p_ = 0.03, while C2 × motivation to protect traditional masculinity interaction was not significant, *B* = 0.01, 95% *CI* = [− 0.04, 0.05], *t*(227) = 0.34, *p* = 0.73. Looking at simple effects revealed that C1 was significant only among men who were less motivated to protect traditional masculinity (− 1SD), *B* = 0.15, 95% CI = [0.04, 0.27], *t*(227) = 2.71, *p* < 0.01, η^2^_p_ = 0.03, revealing that ambivalent sexism in the traditional condition (*M* = 3.67) was significantly higher compared to the feminization condition (*M* = 3.40) and marginally higher compared to the control condition (*M* = 3.50, *t*(227) = 1.74, *p* = 0.08). The control and the feminization conditions did not differ from each other *t*(227) = − 1.01, *p* = 0.31 (see Fig. 1). No other effect reached significance, all *ts*(227) < − 1.41, *ps* > 0.160.^2^

## Discussion

Results of Study 1 confirm our main prediction regarding a difference in sexism between the feminization and traditional norm conditions among men who were less motivated to protect traditional masculinity (henceforth, less traditional men; H1). Simple effects revealed that priming a traditional norm of masculinity increased sexism among less traditional participants, while the feminization condition did not decrease it. Although we did not anticipate this result, these effects are consistent with SRT. SRT suggest that the traditional gender role division is associated with the expectancy of women being more communal and empathic and men being more agentic and dominant. Highlighting a traditional norm of masculinity may have emphasized the traditional gender division and compelled less traditional men to conform to it. Consistent with this reasoning, past research [10] suggests that traditional norms may be challenging for less traditional men because they force them to show conformity to traditional roles, while more feminized norms freed them from this conformity pressure. Finally, the difference between the feminization and traditional norm conditions among more traditional participants (H2) was not significant. We will come back to this unexpected finding in the general discussion. To further explore the effects of men's gender norms on gender-hierarchy-legitimizing ideologies, we ran a second study on a different dependent variable (i.e., masculinism).

### Study 2

Study 2 was designed to replicate the findings of Study 1 with a different dependent variable, i.e., masculinism. Masculinism is an ideology and set of beliefs supporting the idea that masculinity in crisis in the face of the challenge posed by feminist and LGTBI advances. This ideology is based on antifeminism and supports three basic beliefs. The first is gynocentrism, the belief that society privileges women and relegates men to a subordinate position. The second is male victimization, the self-perception that men are victims, and the third is the trivialization of violence against women [8]. Recent reports depict masculinism as a crucial element in the socialization of men and the legitimation of (white) men’s privileged position in the gender hierarchy [45]. The understanding of the reasons that would bring men closer to this ideology is therefore necessary to understand the perpetuation of male hegemony in more subtle forms.

## Method

### Participants and Design

Based on recommendations to recruit at least 50 participants per experimental condition [78, 79], we recruited 182 heterosexual North American men via Amazon’s Mechanical Turk. They were compensated with USD 0.75 for their time. Three participants were excluded from the analyses because they did not provide their consent for the use of their data (*n* = 3). The final sample consisted of 179 heterosexual American men (*M*_age_ = 36.10 years, *SD*_age_ = 10.53 years). A sensitivity power analysis conducted on G*Power for a multiple linear regression model with five predictors, assuming an α of 0.05 and a power of 0.80, revealed that our final sample afforded us enough power to detect a small effect size (*f*^2^ = 0.06). Participants identified as White (56.4%), Asian American (12.3%), Hispanic/Latino (12.3%), Native American (10.1%), and African American (7.8%).

### Independent Variables

*Motivation to protect traditional masculinity* was assessed in the same way as in Study 1 (*M* = 4.52, *SD* = 1.01, α = 0.93).

*Manipulation of the gender norm* was induced as in Study 1, the only difference being that the study was presented as being conducted in a U.S. University to match participants’ national background.

### Measurements

#### Masculinist Beliefs

After being exposed to the manipulation of men’s norm, participants were asked to report their level of agreement with a 20-item scale assessing masculinist beliefs [7]. In line with masculinist concerns, the scale encompasses two sub-dimensions related to men’s identity and status. The identity subscale assesses men’s concerns over men’s role in society (e.g., “Today, men are confused about how to behave”). The status subscale assesses men’s concern about women’s domination over men (e.g., “Nowadays, men are subordinated to women”). The correlation between the two masculinism subscales was strong and positive, *r*(179) = 0.80, *p* < 0.001, suggesting that both subscales measure the same construct. Accordingly, all the items were averaged into a single score, wherein higher scores reflect a higher endorsement of masculinist beliefs (*M* = 4.35, *SD* = 1.34, α = 0.96). The scale has good reliability and predictive validity, and it has been shown to correlate with both hostile and benevolent sexism [46], hostility toward women [65] and rape myth acceptance [74].

#### Experimental Manipulation Check

Acknowledgment of changes in men’s gender norms was assessed as in Study 1. Higher scores reflect a higher perception of men’s feminization (*M* = 4.72, *SD* = 1.32, α =.81).

## Results

As in Study 1, regression analyses were conducted to test our hypothesis. Again, the three levels of the manipulated variable were broken into two contrasts: The main contrast tested the linear effect between the three experimental conditions by opposing the feminization condition to the traditional condition with the baseline-control condition situated in-between; C2 verified the residual variance (C1: − 1 = traditional, 0 = control, + 1 = feminization, and C2: − 1 = traditional and feminization, + 2 = control). As in study 1, we regressed dependent variables on the two contrasts, men’s endorsement of traditional masculinity norms (standardized scores), as well as the interaction between this factor and each contrast (interactions between the two contrasts were not included).

### Experimental Manipulation Check

The analysis revealed a significant main effect of motivation to protect traditional masculinity, *B* = 0.72, 95% *CI* = [0.56, 0.89], *t*(173) = 8.80, *p* < 0.001, η^2^_p_ = 0.31. Overall, men’s gender norms were perceived as becoming more feminine as endorsement of traditional masculinity norms increased. The main effect of C1 was also significant, *B* = 0.27, 95% *CI* = [0.07, 0.47], *t*(173) = 2.64, *p* < 0.01, η^2^_p_ = 0.04. As expected, acknowledgment that men’s gender norms are becoming more feminine was stronger in the feminization condition (*M* = 5.02) than in the traditional condition (*M* = 4.50), with the baseline-control condition (*M* = 4.62) lying between the two experimental conditions. No other effect reached significance, *ts* < 1.91, *ps* > 0.06. Simple effects revealed that the control condition did not differ from the traditional condition (*t*(173) = − 0.68, *p* = 0.50) but differed from the feminization condition (*t*(173) = 2.01, *p* = 0.04).

### Masculinist Beliefs

**Fig. 2:**
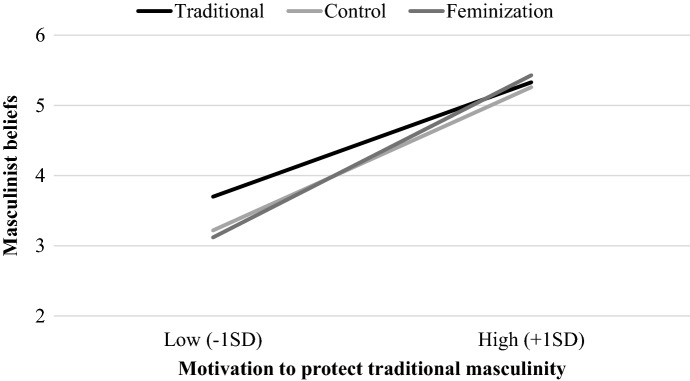
Effects of men’s gender norms on masculinist beliefs as a function of participants’ motivation to protect traditional masculinity (Study 2)

The analysis revealed a main effect of motivation to protect traditional masculinity, *B* = 1.00, 95% *CI* = [0.87, 1.14], *t*(173) = 14.63, *p* < 0.001, η^2^_p_ = 0.55. Overall, endorsement of masculinist beliefs increased as endorsement of traditional masculinity norms increased. This effect was qualified by a significant C1 × motivation to protect traditional masculinity interaction, *B* = 0.17, 95% *CI* = [0.01, 0.34], *t*(173) = 2.00, *p* = 0.047, η^2^_p_ = 0.02, while the C2 × motivation to protect traditional masculinity interaction was not significant, *B* = 0.02, 95% CI = [− 0.08, 0.11], *t*(173) = 0.25, *p* = 0.80. Simple effects revealed that C1 was significant only among less traditional men (− 1SD), *B* = 0.29, 95% CI = [0.06, 0.52], *t*(173) = 2.45, *p* = 0.015, η^2^_p_ = 0.03, so that masculinism was significantly higher in the traditional condition (*M* = 3.70) compared to the feminization (*M* = 3.12) and control conditions (*M* = 3.22). The control and feminization conditions did not differ from each other (*t*(173) = − 0.43, *p* = 0.67; see Fig. 2).^3^ No other effect reached significance, all *ts*(227) < − 1.41, *ps* > 0.160.

## Discussion

Results of Study 2 are consistent with those observed in Study 1 and confirm our main prediction regarding a significant difference between the feminization and traditional norms conditions among less traditional participants (H1). Again, simple effects revealed that priming a traditional norm of masculinity increased masculinism among less traditional participants, while the feminization condition did not decrease it. These findings provide further evidence that traditional norms may be onerous for less traditional men because they are compelled to conform to an ingroup norm that does not reflect their conception of masculinity, whereas more feminized norms liberate them from this ingroup conformity pressure (see [10]). Finally, the difference between the feminization and traditional norms conditions among more traditional participants (H2) was not significant, which is at odds with past research [3, 13, 36, 50, 84].

## General Discussion

Across two studies, we investigated the consequences of the feminization of men’s gender norms (vs. traditional norms) on men’s gender-hierarchy-legitimizing ideologies. According to Precarious Manhood Theory [87, 88] and Social Role Theory [31], we overall expected to observe a significant difference between the feminization and the traditional norms conditions, with a baseline-control condition lying in between. This effect was expected to be moderated by men's motivation to protect traditional masculinity [3, 10, 36, 51, 84]: The feminization condition should decrease gender-hierarchy-legitimizing ideologies among less traditional men (i.e., those who are less motivated to protect traditional masculinity) compared to the traditional norm condition (H1), whereas the feminization condition should increase gender-hierarchy-legitimizing ideologies among more traditional men (i.e., those who are more motivated to protect traditional masculinity) compared to the traditional norm condition (H2).

Both studies consistently supported H1 and showed that the feminization norm condition differed from the traditional norm condition among less traditional men. Interestingly, specific comparisons between the two experimental and the control conditions revealed that priming less traditional participants with a traditional norm of masculinity increased their legitimation of the gender hierarchy, while priming a feminization of men’s gender norms did not influence their legitimation of the gender hierarchy. Overall, these findings are consistent with SRT because the salience of traditional gender roles increases conformity to a traditional view of gender. Moreover, they denote the importance of considering a control condition in order to conclude on the directions of the observed effects. Indeed, based on these results, we could speculate that the traditional norm condition increases less traditional participants' perceptual salience of the intergroup context [83]. Under such circumstances, intergroup salience drives low-committed ingroup members to further seek intergroup differentiation and conform to the prevalent traditional norm [54, 55]. These findings also suggest that traditional norms may be challenging for less traditional men because they are forced to conform to the traditional ingroup norm. Thus, a more feminized norm could free them from the pressure of traditional masculinity and allow them to express attitudes that are closer to their core beliefs (see also [10]).

Both studies consistently showed no evidence for a defensive reaction among more traditional men, which is at odds with H2. Contrary to our expectations based on PMT, exposure to information emphasizing the feminization of men’s norm did not increase more traditional men’s endorsement of gender-hierarchy-legitimizing ideologies. Indeed, and given the precarious status of masculinity, we would have expected the feminization of men’s norm to trigger defensive reactions among more traditional men to reaffirm the gender hierarchy blurred by the alleged feminization. Both studies, however, suggest that more traditional men may be already reaffirming intergroup differences regardless of the experimental condition. Indeed, in Study 1, we observed a lack of difference between the feminization and control conditions on the manipulation check measure suggesting that men perceive their gender group as becoming more feminine. Similarly, in Study 2, we observed a main effect of participants’ motivation to protect traditional masculinity on the manipulation check, suggesting that the more participants are motivated to protect traditional masculinity, the more they perceive their gender group as becoming more feminine. Thus, the findings on the manipulation check measures may inform on the absence of effects of our manipulated variable.

Interestingly, the high scores on the gender-hierarchy-legitimizing ideologies in the three experimental conditions of both studies suggest that traditional men are motivated to reinforce the gender hierarchy regardless of information concerning gender boundaries. This may indicate, first, that traditional men may feel threatened by default and thus are more motivated to protect the gender hierarchy in all circumstances. Second, this may also indicate that previous research failed to detect men's motivation to maintain the gender hierarchy independently of male norms because it has focused solely on men's self-description or attitudes toward deviant ingroup members. Moreover, the content of traditional masculinity norms seemed to encourage the domination of men over women. This may then explain the higher levels of gender-hierarchy-legitimizing ideologies in the three experimental conditions and regardless of their content. Interestingly, this suggests that the mechanisms that operate in the two experimental conditions may differ. While the feminization of the men’s norm threatens distinctiveness, traditional masculinity simply makes the norm of dominance more salient and motivate traditional men to conform to it. More research on this topic is needed.

## Limitations and Future Research Directions

The present research makes an important contribution to our understanding of the dynamic nature of gender norms and the consequences of the evolving role of men on gender relations. At the same time, some limitations need to be mentioned. First, in both studies, participants’ motivation to protect traditional masculinity was assessed at the beginning of the questionnaire, which could have activated a traditional masculinity mindset among participants and influenced the impact of the norm manipulation. However, in both studies, participants overall scored close to the middle of the scale, suggesting that some participants are relatively aligned with traditional masculinity norms, while others are relatively unaligned. Therefore, it is unclear whether assessing motivation to protect traditional masculinity can directly impact the manipulation of the norm in any way. Nevertheless, this potential limitation suggests the need for alternative methods to rule out the possibility that the present results were influenced by the activation of a traditional mindset. For instance, further research could operationalize motivation to protect traditional masculinity by using a false feedback paradigm (e.g., [68]. Furthermore, given that motivation to protect traditional masculinity is related to the support for the status quo [51], future research could assess motivation to protect the status quo using alternative individual differences such as political orientation, social dominance orientation, or system justification.

Second, we did not observe any evidence in support of a defensive reaction among more traditional men (H2). Beyond the theoretical interpretations for the absence of results, some methodological issues may also be accountable. Indeed, our dependent variables are meant to capture the need for differentiation by measuring participants’ attitudes toward the gender hierarchy. In this regard, Jetten et al. [55] observed that defensive reactions aimed at restoring distinctiveness between groups emerge mainly on behavioral measures rather than on judgmental measures (i.e., prejudice; see also [40]). This difference is explained by the purpose that the two measures may serve. Whereas judgmental measures are descriptive in nature and may be sufficient to establish differentiation between groups when it is already granted (i.e., when groups are different), behavioral measures are more suitable for establishing differentiation when groups are similar, thus, when intergroup distinctiveness is threatened. Given that the two dependent variables used in the present research were rather descriptive, one may assume that we were less likely to observe a defensive reaction among traditional men. Future research should consider comparing a judgmental measure to a behavioral measure, such as candidate selection.

Our experimental manipulation check may also raise concerns. Participants had to indicate whether, in their opinion, men’s ingroup norms have become more feminine, which might have highlighted the possibility that they were more or less in agreement with the information provided in their experimental condition. Indeed, manipulation checks can sometimes induce participants to counter-correct the experimental information, leading to unpredicted outcomes [37]. However, both studies introduced the checks at the very end of the questionnaire, which means that the manipulation check could not have impacted the observed results. Nevertheless, and as expected, the results showed that participants exposed to the feminization condition perceived that men are becoming more feminine compared to participants exposed to the traditional condition, clearly indicating that the information impacted participants' personal beliefs. Differences were observed in the comparison between the control- and the two experimental conditions, with Study 1 reporting no difference between the control and feminization conditions but a significant difference with the traditional condition. Conversely, Study 2 found no difference between the control and traditional conditions but a significant difference with the feminization condition. Thus, we can be confident that the information provided in the two experimental conditions was well integrated by our participants, even though we must be careful when interpreting the results of the control condition.

More broadly, our research was the first to demonstrate that a normative change in men’s gender norms may have deleterious consequences for outgroups (i.e., women) without the need for the outgroup itself to be responsible for this social change. However, as the masculinist movements suggest, some men may believe that men are becoming more feminine because of women or because of deviant ingroup members. For now, we cannot comment on the mechanisms behind these effects. Indeed, our results do not inform us whether the norm feminization is perceived as a change derived by ingroup members or outgroup members, and whether the perceived norm feminization is actually liberating for less traditional men and threatening for more traditional ones. In this regard, future research should build on our findings to investigate the motivations underlying the observed effects.

## Implications and Outlook

Gender norms and gender-hierarchy-legitimizing ideologies are both among the most important factors predicting gender inequality [85], which remains one of the most important and persistent issue in Western societies [90]. This research underpins research on toxic masculinity [2], and warns on the harmful effects that traditional masculinity may have on gender relations. Indeed, we demonstrated that men who, by default, are committed to more inclusive values conform to traditional masculinity when is salient and embrace it by reinforcing the gender hierarchy. More worryingly, a traditional norm of masculinity does not seem to reassure more traditional men. Conversely, more traditional men keep their defenses high even when intergroup differentiation is granted by reinforcing the gender hierarchy and affirming their dominance over women. In these circumstances, it is important to understand how to lower men’s defenses in order to encourage them to commit to gender equality. Overall, our research suggests that changes in men’s gender norms may not always decrease men’s endorsement of gender-hierarchy-legitimizing ideologies. Rather the opposite: Our research warns of the harmful effects of traditional masculinity on gender relations and indicates that even less traditional men may be compelled to conform to an ideal of a traditional man in contexts characterized by traditional norms of masculinity.

## Conclusions

The present findings highlight the importance of examining the evolving role of men with regard to gender relations and the maintenance of the gender hierarchy. Two studies showed that less traditional men are more accepting of ideologies that legitimize male dominance over women when confronted with traditional norms of masculinity. Conversely, more traditional men appear to be non-sensitive to information about changes in their gender norms while maintaining high levels of gender-hierarchy legitimization.

## Footnotes

1 Contrast analysis allow us to test a specific hypothesis when a variable has more than two modalities, without arbitrarily dividing the effect of the manipulated variable (see [16, 39]).

2 In order to examine whether the observed results varied as a function of the hostile versus benevolent sexism subscales, we conducted a repeated-measure (mixed) ANCOVA. The two subscales were introduced in the analysis as a within-subjects factor. We also included participants’ endorsement of traditional masculinity (standardized scores), the two contrasts related to the experimental manipulation, as well as all the interaction between these factors. This analysis did not reveal any significant interaction including the within-subjects factor, *F*(1, 227) < 3.52, *p* > .062, indicating a similar pattern of results for both subscales.

3 Despite both masculinist subscales were strongly correlated, we also examined whether the results differed across the two subscales. Thus, we conducted a repeated-measure (mixed) ANCOVA in which, in addition to the same predictors included in the main regression analysis, we included the two masculinist beliefs subscales as a within-subjects factor. No interaction including the within-subjects factor was significant, *F*(1,173) < 0.12, *p* > .725, indicating a similar pattern for both subscales.
